# How to Ensure Referral and Uptake for COPD Rehabilitation – Part 2: A Case of Integrated Care on How to Translate Findings of Cross-Sectorial Workflow to Improve Cross-Sectorial Rehabilitation

**DOI:** 10.5334/ijic.5503

**Published:** 2021-03-02

**Authors:** Lars Morsø, Michael Skriver Hansen, Anette Brink, Mette Thams, Bettina Ravnborg Thude

**Affiliations:** 1 Open Patient data Explorative Network (OPEN), Region of Southern Denmark, DK; 2 Department of Clinical Research, University of Southern Denmark, Odense, DK; 3 Health Department, Municipality of Sønderborg, DK; 4 Department of Medicine, Hospital Sønderjylland, Sønderborg, DK

**Keywords:** cross-sectorial referrals, simplification, relational coordination, network groups

## Abstract

**Background:**

Patients with chronic obstructive pulmonary disease (COPD) can greatly benefit from rehabilitation initiatives, but referral to rehabilitation is sparse. Before we initiated activities to ensure hospital referrals for prevention initiatives at the municipality, we investigated referral patterns and relevant factors in the cross-sectorial workflow.

**Objective:**

To ensure referral to municipality COPD rehabilitation, by simplifying the referral procedures, and by facilitating relational coordination across the two health care settings.

**Methods:**

We simplified the referral procedure by initiating all referrals to contain standard wording, all send to the same electronic location, and assuring that all patients were referred to the same initial interview. We facilitated cross-sectorial relational coordination by establishing local- and cross-sectional network groups. We monitored the network groups, and send questionnaires to obtain knowledge of network activities. We used indicators to measure the cross-sectorial quality and questionnaires to measure the patient experienced quality.

**Results:**

We detected flaws in the referral system that meant that several referrals were neglected. Based on knowledge and experiences the networks called for adjustments. This led to adjustments in patient inclusion and data collection.

**Conclusion:**

We succeeded in simplifying referral procedures and facilitated cross-sectorial relational coordination. We had to make ongoing adjustments of procedures, information, content, population and data infrastructure.in simplifying referral procedures and facilitated cross-sectorial relational coordination. We had to make ongoing adjustments of procedures, information, content, population and data infrastructure.

## Introduction

### Summary of “How to ensure referral and uptake for COPD rehabilitation – Part 1: Disentangle factors in the cross-sectorial workflow to understand non-referral to rehabilitation”

Part 1 of the study used a functional resonance analysis method (FRAM) [1] to understand why most patients are not referred to rehabilitation and to capture relevant and important factors in the cross-sectorial workflow of patients with chronic obstructive pulmonary disease (COPD). This aimed to identify and analyse how patient pathways and daily workflow interact, and which conditions influence the collaboration between municipality and hospital. The data from the FRAM created the basis for a workshop for employees, patients and leaders where the findings were discussed. From the workshop discussions, differences were identified in the understanding of the concept of rehabilitation, and in the language and approaches in terms of diagnosis and functionality across the healthcare system from hospital to municipality. The workshop revealed a complicated referral pathway and variation in the content of referrals, as well as a lack of knowledge and information across healthcare sectors that complicated the cross-sectorial collaboration. Overall, workshop participants stated that cross-sectorial collaboration would be easier if they knew who is “in the other end of the line”, and they suggested activities to develop and coordinate the relations.

### Problem description

Patients with COPD can greatly benefit from rehabilitation initiatives [2], but referral for rehabilitation is rare [3]. Challenges for encouraging patients to participate in rehabilitation include problems with the handover of patients between healthcare sectors and problems related to establishing individualised rehabilitation programmes in municipalities. Successful solutions for these challenges are sparsely described in the literature [4,5].

An earlier attempt to counter challenges related to increased cross-sectorial referrals, uptake and completion of rehabilitation for COPD patients failed [6]. Therefore, new approaches are needed to avoid another failed attempt to improve cross-sectorial rehabilitation for patients with COPD.

To investigate how the results from part 1 of our study could be developed to improve practice, we needed to translate the statements from the workshop and the findings from the analysis of the workflow into specific areas of improvement. To ensure that initiatives lead to quality, studies have emphasised that the launched processes are monitored and information is fed back into improvement programmes [7,8]. Another recommendation to succeed in improving practice is managerial involvement. According to Grol and Wensing [9], managerial anchoring is essential in implementation of quality of improvement.

Subsequently, stakeholders from the municipality and the hospital, including management and staff, agreed to initiate activities to ensure that the hospital would refer more patients to rehabilitation initiatives at the municipality. Based on the findings from the first part of the study, the improvement initiatives focused on two improvement activities: (i) simplifying the referral procedure and (ii) increasing the relational coordination across sectors.

### Objective

The overall objective of this study was to describe and analyse how to translate our findings from the first part of the study to ensure referral and uptake for COPD rehabilitation. Using an action research approach, this part of the study analysed the process of simplifying referral procedures and facilitating cross-sectorial relational coordination.

## Methods

### Description of practice and the rationale behind the development/implementation of the intervention

#### Simplifying referrals

Part 1 of the study showed that the hospital and municipality used different wording (e.g., diagnosis versus level of function) and that staff at the hospital lacked knowledge on how the referral works, where to send it, and rehabilitation offers in the municipality. The study showed that to be able to refer patients to rehabilitation, the clinician needed to know what to refer the patient to, how to complete the referral and where to send it. Clinicians at the hospital struggled to keep track of all municipality rehabilitation services, which made it hard to know where and what to refer the patient to.

#### Relational coordination

To align the conceptual understanding of rehabilitation and minimise differences in diagnostic language and approaches, the hospital and the municipality decided to develop and coordinate the cross-sectorial clinical relations. A cross-sectorial steering group including independent researchers was formed to initiate the project. The steering group hypothesised that better relational coordination would contribute to increased referrals, better patient pathways and improved patient satisfaction. The first step was to acknowledge the need for a higher level of relational coordination. The second step was to ensure the attainment of that higher level. Multidisciplinary meetings are one way to develop relational coordination [10].

Relational coordination is “a mutually reinforcing process of communicating and relating for the purpose of task integration” [11, p.302].

It covers two themes involving staff: (i) good relations, meaning shared goals, shared knowledge and mutual respect, and (ii) good communication, meaning frequent communication, timely communication, accurate communication and problem-solving communication [11]. According to Havens et al. [12, p.503], relational coordination enables “employees to more effectively coordinate their work with each other, thus pushing out the production possibilities frontier to achieve higher-quality outcomes while using resources more efficiently”.

This approach is also known in other industries depending on a high level of quality and security, such as the aviation, nuclear power and shipping industry [13]. An increased level of relational coordination between professionals has in other settings been shown to produce better clinical results and increased patient satisfaction [14,15].

At the workshop, it was discussed what specific actions could be instigated to support and improve the relational coordination. This led to the establishment of local network groups in each organisation and a cross-sectorial network group. The purpose of the networks is to discuss relevant subjects, to plan activities to improve the knowledge of relevant participants, and to develop processes and work procedures to create better patient pathways. Further, the networks aim to allow the hospital staff to obtain updated knowledge of the rehabilitation offers at the municipality. The management from the hospital and the municipality appointed the participants. The local and cross-sectorial network groups consist of nurses and physicians from the hospital and the relevant staff from the municipality, including clinicians and the relevant managers from both sectors.

### Data collection

**Figure 1:**
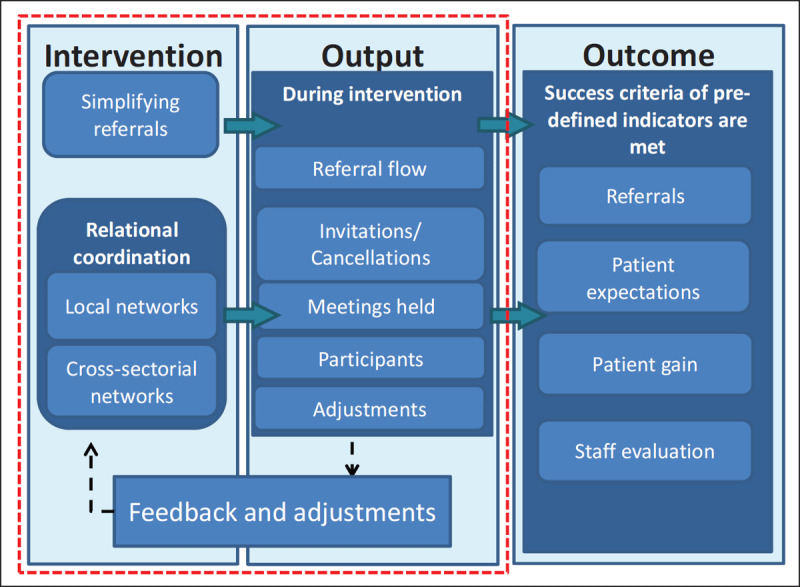
Conceptual model of the intervention.

To conduct our action research, we need insights on how the alteration of the referral pathway, changed content of the referrals and establishment of local/cross-sectorial networks affect the referrals. This is achieved by monitoring the process and feedback information in the study. By application of this feedback mechanism, the initiatives mirror an iterative PDSA (Plan, Do, Study, Act) process [8] with several adjustments (see ***Figure 1***). The continuously collected data and feedback information are discussed at network meetings and steering group meetings, and in dialogue at both management and staff levels.

In this part of the study, the findings of the ongoing improvement process are reported. The results of the outcome indicators, and patient and clinician questionnaires, will be reported in a future outcome article.

Based on feedback from the involved parties, the cross-sectorial steering group decided that the network groups would meet several times a year. To ensure that the network meetings are held, that the participating clinicians attend and that the quality of the meeting is in line with the outlined intentions, prepared agendas before and summaries after each network meeting are required, and each meeting that is carried out is logged in a calendar. The researchers from the steering group continuously monitor and analyse these data and discuss findings at the local/cross-sectorial network meetings and steering group meetings. To monitor the relational coordination, a researcher participates in the network meetings to observe the development of shared goals, shared knowledge, mutual respect, frequent communication, timely communication, accurate communication and problem-solving communication.

None of the primary investigators are involved in daily clinical routines, but throughout the study, they are in close contact with representatives from the staff through meetings in cross-sectorial and local network groups, and with leaders from both setting through steering group meetings.

## Context

The project started on 1 April 2019 and has a duration of 2 years. The setting is the cross-sectorial pathway of the Danish healthcare, consisting of hospital and municipality. Both are financed by taxes and therefore free of charge for the individual patient, and are the main collaborators in our population. We chose to focus on the referral and uptake across sectors, as prior registry data indicated that only few referrals from hospitals to municipality rehabilitation were being made. The hospital is a medium-sized regional facility with approximately 240 beds and includes the specialty of COPD in the medical department. The hospital covers several municipalities, but for the project, we chose to focus on one municipality with 74,198 inhabitants. The project was primed by prior collaboration between the two parties in other areas. From January 2018, the municipality has managed all rehabilitation and prevention initiatives for patients with COPD.

## Ethical considerations

We based participation in the project on informed consent. All patients in the study receive verbal and written information regarding the study. Patients can withdraw consent for participation at any time and still receive rehabilitation in concordance with the usual municipal standards.

For data collection, we use the participants’ e-box, which is a secure mailbox system for Danish citizens. All citizens use this email to receive their correspondence from the public authorities. The participants access these emails by using their unique three-point log-on system as security. Our established electronic REDCap^®^ database (REDCap 9.1.15 - © 2020 Vanderbilt University) uses this system, and we are able to send written consent forms and all questionnaires electronically to the participants’ secure mailboxes. This correspondence requires verbal patient consent, which we obtained in conjunction with the initial interview at the municipality.

By request, the Scientific Ethics Committee of Southern Denmark stated no need for ethical approval for this project. The Danish Data Protection Agency (file no. 15/3321 and 19/3451) approved the study. We store personal information according to all rules of the Danish Data Protection Agency. Data management is conducted according to the rules of the Region of Southern Denmark at the department of OPEN (Open Patient data Explorative Network).

## Results

The hospital and the municipality have simplified the referral procedure on several levels. One simplification is the direction of all referrals to the same main electronic mail location, which simplifies the process for the hospital clinicians and ensures that no referrals are lost because of mailing errors. We therefore expected to see an increase in referrals. However, the number of referrals from the hospital remained low compared with the expected amount due to the prevalence of COPD. It turned out that the municipality digital system needed upgrading to be able to receive and read referrals from the hospital. This flaw was only detected because of the project and meant that several citizens were not rehabilitated in that period. After adjustment and upgrading, the referrals are accessible and the number increased slightly.

The small increase of referrals was still not considered sufficient, and the number of referrals sent by the hospital did not match the number registered in the project or the number registered by the municipality. Examining the referrals sent from the hospital, we found that the shared decision of having all patients referred for an initial interview at the municipality and letting the municipality, clinician and patient decide on the service needed was not complied with. Hospital clinicians still sent patients to the service that they considered relevant for the patients. The consequences were that patients were not included in the study, and more importantly, they potentially did not receive services they might have benefited from. It turned out that there were several reasons for this lack of compliance.

Information with regard to referring patients to one mutual service (the interview) had not been communicated at all relevant units at the hospital. Further, clinicians at the hospital wanted to “help” clinicians in the municipality by providing as much information as possible, despite the agreement that the wording of the referrals should only be a minimum standard set of information determined by mutual understanding at earlier network meetings. It also turned out that the diagnosis of COPD was requested to identify and handle the referral so that it ended at the right electronic location. Finally, we discovered that the municipality in parallel with the project had changed their overall procedures for initial interviews. Despite the agreement of referral to an individual interview for all patients, not all were offered this interview. In the project, we were not informed of this change.

These findings prompted the cross-sectorial network to discuss how to ensure dissemination of information among clinicians at the hospital units. Local champions and experienced doctors are now involved to spread the information of the new procedures in their individual wards. The wording of referrals showed that participants from the hospital and municipality did not have the same understanding of the words “prevention” and “rehabilitation” used in the referrals. To overcome any confusion, the wording was adjusted to use only the word “prevention” as it more precisely reflects the municipality activities of preventing exacerbations of the illness to increase the quality of patients’ everyday life; on the other hand, municipality clinicians had to integrate the COPD diagnosis coding into their everyday language. Finally, the changes of the initial procedures in the municipality were discussed at a steering board meeting, and it was realised that the changes were counterproductive for the cross-sectorial activities in the study. After an executive decision in the municipality, the changes were stopped, and all patients are now offered an initial interview.

In the planning process of having all patients referred for an initial interview, the network decided that staff at the hospital should inform the patients about the project and should motivate the patients to accept the rehabilitation offer from the municipality. However, this did not work in the everyday setting as the hospital staff had too little time in dialogue with the patients to handle these tasks. Therefore, adjustments were made to handle information when the patient arrives at the initial interview at the municipality. Municipality staff now explain about the research project and motivate patients to participate in rehabilitation. This procedure seems to work for all parties.

The organisational part of the intervention was the specific initiative of establishing local and cross-sectorial networks. All participants appointed to the local and cross-sectorial networks accepted the appointment. No meetings have been cancelled; agendas are available at a secure SharePoint^®^ site, and minutes are available after every meeting. After meetings during the first 6 months, the network groups called for adjustments based on new knowledge and experiences. Prior to the intervention, it was decided that the project should only include patients recently diagnosed with COPD. However, as the network groups started their dialogues and discussed patient recruitment, it was clear that it did not make sense to include only recently diagnosed patients. Consequently, the steering group decided to include all patients diagnosed with COPD. The network also called for managerial participation, which has been implemented in the networks. Over time, the frequency of the network meetings has decreased, but it was stated that meetings are still useful and are held when needed. Overall lessons learnt are shown in ***Table 1***.

**Table 1:** Implementation of findings from part one and lessons learnt during the process.


IMPROVEMENT INITIATIVE	LESSON LEARNT

Simplifying referrals	

Central mail location	Monitoring of the functionality of digital systems is required Ongoing adjustments of referral content Diagnostic coding ensuring recognition of referral Dissemination of information to relevant referrers

Patients referred for similar procedure (initial interview)	Awareness of competing interests interfering with study procedures Several re-adjustments regarding procedures of the initial interview Devoting time for dialogue with patients

Referral phrases/wordings	Network discussions of how to understand different referral wordings

Relational coordination	

Local network groups Local and cross-sectorial networks	Appointing relevant staff members (champions and opinion makers) Closer contact with the decision-makers during the process Re-visiting agreements Re-visiting perception of information


We developed a data collection infrastructure in the project, but the described adjustments to the project affected this data collection. Therefore, we needed to adjust the inclusion module of the database to capture the Global Initiative for Chronic Obstructive Lung Disease (GOLD) classification (which divides COPD into four stages (1–4) to determine the degree of the disease) of the patients [18] and the referral site (hospital, general practitioner, self-contact). This adjustment allows us an overview of the burden of disease and referral sources.

## Discussion

We set out to investigate whether it was possible to simplify referral procedures and facilitate cross-sectorial relational coordination to increase the number and quality of referrals to municipality COPD rehabilitation. We used knowledge from the first part of the study (FRAM and workshop material) that revealed differences in how to conceptualise rehabilitation, and the articulation of diagnosis and functionality used across the healthcare system from hospital to municipality. Moreover, we found a lack of knowledge and information across healthcare sectors that complicated the cross-sectorial collaboration.

Detangling specific factors from local contexts has earlier proved important [7,16]. By doing this, improvement work becomes relevant for frontline clinicians to implement. First, all referrals are directed to one mail allocation. Earlier studies have found that referrals sent to several locations have a tendency not to be activated, whereby the patient is not rehabilitated [3]. Complicated referral procedures are viewed as a barrier to cross-sectorial rehabilitation [17]. Data transparency across healthcare is therefore important for increasing referrals to rehabilitation. In this study, the organisational challenges are considerable. These challenges have to be addressed repeatedly, and close monitoring is essential to ensure that referrals are not lost in the transition from hospital to municipality. Second, all patients are specifically referred for an initial interview at the municipality to plan rehabilitation individually. This is done because patient adherence has been shown to be problematic in COPD rehabilitation [18], and a way to increase participation is to individualise rehabilitation [19]. Another barrier for adherence is motivation. Poor perceived health, expected benefits and practical considerations concerning rehabilitation are considerable barriers for adherence [20]. By referral for an initial interview, individual patient needs are revealed and practical considerations can be addressed. Clinicians who are involved in the rehabilitation provide information, and staff at the hospital are released from having to motivate patients to participate in rehabilitation services about which they are poorly informed. Although this referral procedure was agreed upon from all study participants, adjustments during several improvement circles had to be made. Poor information and opaque pathways had to be unravelled before referrals ended up as intended. Third, the wording of the referrals has been simplified. Studies have revealed a number of barriers for the implementation of scientific evidence into practice [9], such as professionals’ attitudes, and organisational and political contexts [9]. By using the same language and phrases in referrals, clinicians are able to relate to other parties involved in cross-sectorial collaboration, and clarification is obtained on organisational and political obstacles. In our study, the involved parties attempted to simplify the referrals by including only the COPD diagnosis coding and the phrasing “initial interview”. Although simple, this proved problematic. Most clinicians wanted to ensure that no information was lost, and included substantial details in the referrals. The steering group meeting dialogues with the clinicians showed that they took personal pride in assessing and referring patients to services that they considered appropriate. They felt that their clinical autonomy was affected and their decision-making undermined. This is in line with earlier findings [18] and raises questions regarding who is responsible for the clinical decision.

To facilitate cross-sectorial relational coordination, the hospital and the municipality formed network groups. Facilitation of relational coordination has been shown to increase quality and efficiency [11,14,15]. During the process, we needed to adjust the composition of the groups to include leading staff. This is in line with recommendations from the literature that suggest that management support is a crucial part of implementation processes that lead to change [9,21]. To date, we do not have sufficient data to report whether the relational coordination has improved the referral frequency and patient uptake in the municipality. The participants in the study state that even though the first few network meetings have contributed to increased knowledge of cross-sectorial collaboration, there are considerable challenges in the development of shared goals, frequent communication, timely communication, accurate communication and problem-solving communication.

In the study, we developed a measurement structure to capture the changes of the planned cross-sectorial activities. In evaluation of planned changes, a variety of factors should be taken into account, including the ability to measure both quantitative outcome parameters and local factors of importance [22]. The recommended use of programme theory [16] has been used in the study to plan a measurement structure that embraces multiple parameters. In this part of the study, we have not reported these data, and the ongoing data collection will reveal whether further adjustments will have to be made. This data collection will take place next year, and in future work, we will report whether standards and indicators have been fulfilled. Further, we will supplement the quantitative reporting with patient and clinician interviews to qualify our findings.

Embedding quality improvement projects in more rigorous research designs has been suggested in earlier international research [16,23,24] and may be expanded to other quality improvement projects in the future. Problems regarding cross-sectorial referral and the difficulties of coordinating rehabilitation across healthcare boundaries have earlier been highlighted in other settings [25,26]. Hence, the complexity across healthcare boundaries between hospital and municipality seems to be universal, and the action research approaches from this study to align referrals and improve relational coordination to increase cross-sectorial referrals may certainly be applicable outside the Danish healthcare system. In the present study, we expect to expand the study to include three more municipalities in the uptake area of the hospital as a way to upscale the study.

## Conclusion

In this part of the study, we have introduced an iterative action research approach to improve cross-sectorial referral to rehabilitation by simplifying referral procedures and facilitating cross-sectorial relational coordination by creating network groups. We are utilising feedback to make ongoing adjustments in organisational procedures, information, referral content, population and data infrastructure. Adjustments in the organisation of the network groups have also been warranted. We found initial flaws in the referrals system, and needed to align information, procedures and content of referrals to make the referral structure applicable across sectors. The impact of future research might be improved by routine application of rigorous research designs.

## Appendix 1

Overall overview of project components, indicators, registrations, measurement intervals and success criteria.

**Table:**

INTERVENTION	INDICATOR	REGISTRATION	MEASUREMENT INTERVAL	SUCCESS CRITERIA

Local COPD network	Number of meeting invitations	All invitations including agendas are stored at the SharePoint site of the project	Quarterly	All invitations must include a structured agenda

	Number of meetings that have been held	All summaries from the meetings are stored at the SharePoint site of the project	Quarterly	Minimum three meetings per year have to be held

	Number of cancellations	Cancellations are registered in the calendar at the SharePoint site	Quarterly	None of the planned meetings are cancelled

	Number of participants	Extracted from summary at the SharePoint site	Quarterly	Full participation at all meetings

	Knowledge of the network	Questionnaires are sent electronically to relevant employees through REDCap Randomly selected interviews	At the end of the intervention Interviews and questionnaires at 3 and 12 months (24 months)	Minimum 80% have knowledge of the local network, and interviews indicate importance

Cross-sectorial COPD network	Number of meeting invitations	All invitations including agendas are stored at the SharePoint site of the project	Quarterly	All invitations must include a structured agenda

	Number of meetings that have been held	All summaries from the meetings are stored at the SharePoint site of the project	Quarterly	Minimum three meetings per year have to be held

	Number of cancellations	Cancellations are registered in the calendar at the SharePoint site	Quarterly	None of the planned meetings are cancelled

	Number of participants	Extracted from summary at the SharePoint site	Quarterly	Full participation at all meetings

	Knowledge of the network	Questionnaires are sent electronically to relevant employees through REDCap Randomly selected interviews	At the end of the intervention Interviews and questionnaires at 3 and 12 months (24 months)	Minimum 80% have knowledge of the local network, and interviews indicate importance

Patient experience	Indicator	Registration	Measurement interval	Success criteria

Expectations	Number of patients whose expectations are fulfilled	Questionnaires are sent electronically to participants through REDCap prior to and after the rehabilitation	Prior to the initial visitation in the municipality and after rehabilitation	Minimum 80% of the participants indicate that rehabilitation has fulfilled their expectations

Gain of rehabilitation	Number who found rehabilitation worthwhile	Questionnaires are sent electronically to participants through REDCap after rehabilitation	After rehabilitation	Minimum 90% of the participants indicate that rehabilitation was worthwhile

	Proportion of patients with increased quality of life	Questionnaires are sent electronically to participants through REDCap after rehabilitation	After rehabilitation	Minimum 90% of the participants indicate that rehabilitation has increased their quality of life

	Proportion of patients who obtained predefined goals of rehabilitation	Extracted and analysed from participants’ initial goalsetting in the municipality	At the end of the intervention	Minimum 80% of the participants indicate that they have obtained their predefined goals of rehabilitation

	Patient experiences with rehabilitation	SPOT questionnaire Randomly selected interviews	Quarterly interviews	Interviews to qualify how referral, visitation and rehabilitation are experienced

Quality of the cross-sectorial pathway	Indicator	Registration	Measurement interval	Success criteria

Outcome of the intervention	Number of referrals	Registry data from hospital and municipality	Quarterly	Minimum 90% of patients admitted/diagnosed with an episode of COPD referred to municipality visitation

	Number of patients with only the initial visitation	Registry data from hospital and municipality	Quarterly	Proportion of patients only seen at initial visitation increases by maximum 10%

	Staff evaluation of the intervention	Randomly selected interviews	At the end of the intervention	Interviews to indicate if the intervention has created more focus on rehabilitation
